# Periprosthetic Fractures After Total Knee Arthroplasty—A Two-Center Study and Systematic Review of the Literature

**DOI:** 10.3390/medicina62091727

**Published:** 2026-09-08

**Authors:** Piotr Sypień, Daniel Jaglarz, Jan Gralewski, Ewa Tramś, Witold Bołtuć, Rafał Kamiński, Dariusz Grzelecki

**Affiliations:** 1 Department of Trauma and Orthopedic Surgery, Sebastian Petrycy Health Care Facility, Szpitalna 1, 33-200 Dabrowa Tarnowska, Poland; piotr.sypien@gmail.com (P.S.); ortopediaopis@zozdt.pl (W.B.); 2 Department of Orthopedics and Trauma Surgery, Ceynowa Hospital in Wejherowo, Jagalskiego 10, 84-200 Wejherowo, Poland; jaglarzd@gmail.com; 3 Department of Orthopedics and Reconstructive Surgery, Centre of Postgraduate Medical Education, Adam Gruca Orthopedic and Trauma Teaching Hospital, Konarskiego 13, 05-400 Otwock, Poland; jan.gralewski@gmail.com; 4 Department of Orthopedics and Muscoskeletonal Trauma, Centre of Postgraduate Medical Education, Adam Gruca Orthopedic and Trauma Teaching Hospital, Konarskiego 13, 05-400 Otwock, Poland; ewatrams@gmail.com (E.T.); raf.kaminski@gmail.com (R.K.)

**Keywords:** total knee arthroplasty complications, periprosthetic fracture, revision total knee arthroplasty, osteosynthesis

## Abstract

*Background and Objectives*: Periprosthetic fracture is a complex and urgent condition in orthopedic surgery. It requires an individual approach to every patient, and treatment is associated with high risk of complications. *Materials and methods*: Fifty-six patients treated due to periprosthetic fracture (PPF) after total knee arthroplasty were retrospectively analyzed according to surgical protocol, treatment time, and complication rate. The results were compared to the available literature. *Results*: Almost all patients (91%) were treated surgically—revision total knee arthroplasty or osteosynthesis. A therapeutic success was achieved in 81% of all cases. Replacement of the femoral component with the semi-constrained type of implant was performed most frequently during revision total knee arthroplasty. In PPFs of the femur, destabilization of fixation was observed mainly in type II according to the Lewis–Rorabeck classification. *Conclusions*: Despite the complexity of the problem, treatment of periprosthetic knee fractures can be effective. Provided that the implant remains stable, osteosynthesis is a good treatment option for PPF of the femur. In revision total knee arthroplasty, implants with a higher level of restriction are usually used to ensure better knee joint stability.

## 1. Introduction

With an increase in the number of total knee arthroplasties (TKAs) performed in recent years, the number of revision TKAs due to different complications is constantly growing [1]. Among the most serious complications after TKA are periprosthetic fractures (PPFs) of the knee. Femoral PPFs are the most common, with an incidence rate of 0.3–2.5%; followed by PPFs of the tibia (0.4–1.7%), while patellar fractures after TKA occur on average in 1.19%, and in 99% of cases in patients undergoing patellar replacement [2,3]. Fractures following the placement of revision knee implants occur ten times more frequently [2,4].

However, this frequency depends on several factors, such as the timing of the fracture (intraoperative, postoperative) and whether the PPF is complex, and due to different fracture patterns and usually poor bone quality, treatment requires an individual approach to every patient. Despite many efforts and the development of various therapies, there is still a high rate of failure and complications, including a high mortality rate [5,6]. Important factors in treatment planning include the location and morphology of the fracture, the quality of the bone and soft tissue, implant fixation to the bone, and the type of primary implant [4,7].

Currently, it is admitted that conservative treatment (CON) of PPF should be used in maximally rare circumstances, which are primarily related to the patient’s ineligibility for surgical intervention due to a poor general condition. Use of immobilization in PPF, even if initially assessed as non-displaced fractures, has a significant risk of bone malunion or nonunion, and limited function of the limb preceded by prolonged immobilization [2]. The osteosynthesis of PPF is varied, and includes both close reduction with internal fixation (CRIF), and open reduction with internal fixation (ORIF), as well as revision total knee arthroplasty (rTKA) [7]. Both low compression plates (LCPs) and intramedullary nailing (IMN) are widely used in fracture stabilization. The LCP allows direct fracture repositioning and angle-stable fixation, whereas the use of IMN results in less tissue destruction during intervention. However, IMN raises concerns about the quality of fracture stabilization, especially when the main fracture gap is close to the epiphysis of the bone. In comparison, intensively developed minimally invasive techniques for minimally invasive plate osteosynthesis (MIPO) allow fracture reduction with fewer undesirable effects associated with extensive tissue preparation. On the other hand, rTKA allows rapid restoration of anatomical relationships and quick start of rehabilitation, but is associated with greater surgery, replacement of prosthesis components, and bone resection. Simultaneously, rTKA with implants with extensions allows for the successful treatment of fractures in the proximal tibial epiphysis distal to the primary knee prosthesis [8]. Complementing rTKA is the use of a megaprosthesis of the knee, which provides immediate stability and weight transfer [9]. The role of external stabilization is marginal and most often used as a temporary treatment [10]. Although there is some consensus in the literature regarding PPF types, there is still a need to systematically study them and compare treatment outcomes to further flag remaining discrepancies. In addition, the development of modern, minimally invasive surgical techniques offers the potential to improve clinical outcomes.

Therefore, the main objective of this study was to perform a detailed radiological, clinical, and functional evaluation of PPF in both the femur, tibia, and patella, and to compare these results with existing findings.

## 2. Materials and Methods

### 2.1. Cohort Analysis

This analysis of clinical and radiological results of treatment of PPF after TKA was conducted among patients treated between 2014 and 2022 in two orthopedic centers in Poland. The study was conducted according to the World Medical Association Declaration of Helsinki and was approved by the local Scientific Research Board of Institution (No. 41/2026). The inclusion criterion for the study was the occurrence of a fracture of the femur, tibia, or patella in the area of the knee prosthesis. The exclusion criteria were fractures after unicompartmental knee arthroplasty and megaprostheses, fractures associated with the use of surgical navigation technology, and periprosthetic joint infections and inter-prosthetic fractures (e.g., between knee and hip implants). Factors related to the type of injury, location of the fracture, treatment method, and radiological and clinical outcomes were evaluated. Femur fractures were classified according to the Lewis–Rorabeck classification (LR, Table 1), while tibia fractures were classified according to the Felix classification (FX, Table 1) [11,12].

During the observation period, 71 patients were hospitalized at our medical centers due to PPF and met the inclusion criteria. Twelve patients were excluded from the study because they met the exclusion criteria. Of the remaining group of 59 patients, 3 were excluded due to incomplete medical records. Finally, 56 patients were included in this retrospective observational study.

All study patients were hospitalized and received follow-up care in outpatient clinics for 2–5 years (median: 3 years). Plain X-rays in anteroposterior (AP) and lateral projections were used to assess the fracture type and the progress of bone healing. Data were collected on comorbidities, type of injury and treatment, procedure time, length of hospitalization, and early and mid-term complications. Body mass index (BMI) above 30 kg/m^2^ was considered obese. All patients received complete treatment, including anticoagulant pharmacoprophylaxis (40 mg of enoxaparin s.c. per day) and rehabilitation. Patients undergoing surgery received preoperative preparation and perioperative antibiotics—2 g (3 g over 120 kg body mass) cefazoline i.v. depending on body weight, followed by two doses of 1 g cefazoline i.v. at 8 and 16 h post-operation.

Due to the long observation period, the retrospective nature of the study, and the presence of several orthopedic departments within two medical centers, patients were operated on by different surgical teams within the respective hospital departments.

Therapeutic success (cure) was characterized by no pain at the injury site, radiographic evidence of bone union during follow-ups with regard to patients with fracture reduction, functional joint movement without pain, and no revision surgery.

Statistical analyses were conducted using Microsoft Office 2016 Excel^®^ (Microsoft Corporation, Redmond, WA, USA) and PQStat 1.8.6^®^ (PQStat, Poznan, Poland). Categorical data were summarized using frequency (n), percentages (%), and interquartile range (IQR). The Shapiro–Wilk test was used to check the normality of the distribution. The Student’s *t*-test was used for parametric, and the Mann–Whitney U test for nonparametric. The Fisher test and risk ratio (RR) were used to check differences for dichotomous variables. *p* ≤ 0.05 was assumed to be statistically significant, and RR was estimated with a 95% confidence interval (CI).

### 2.2. Systematic Review

**Figure 1:**
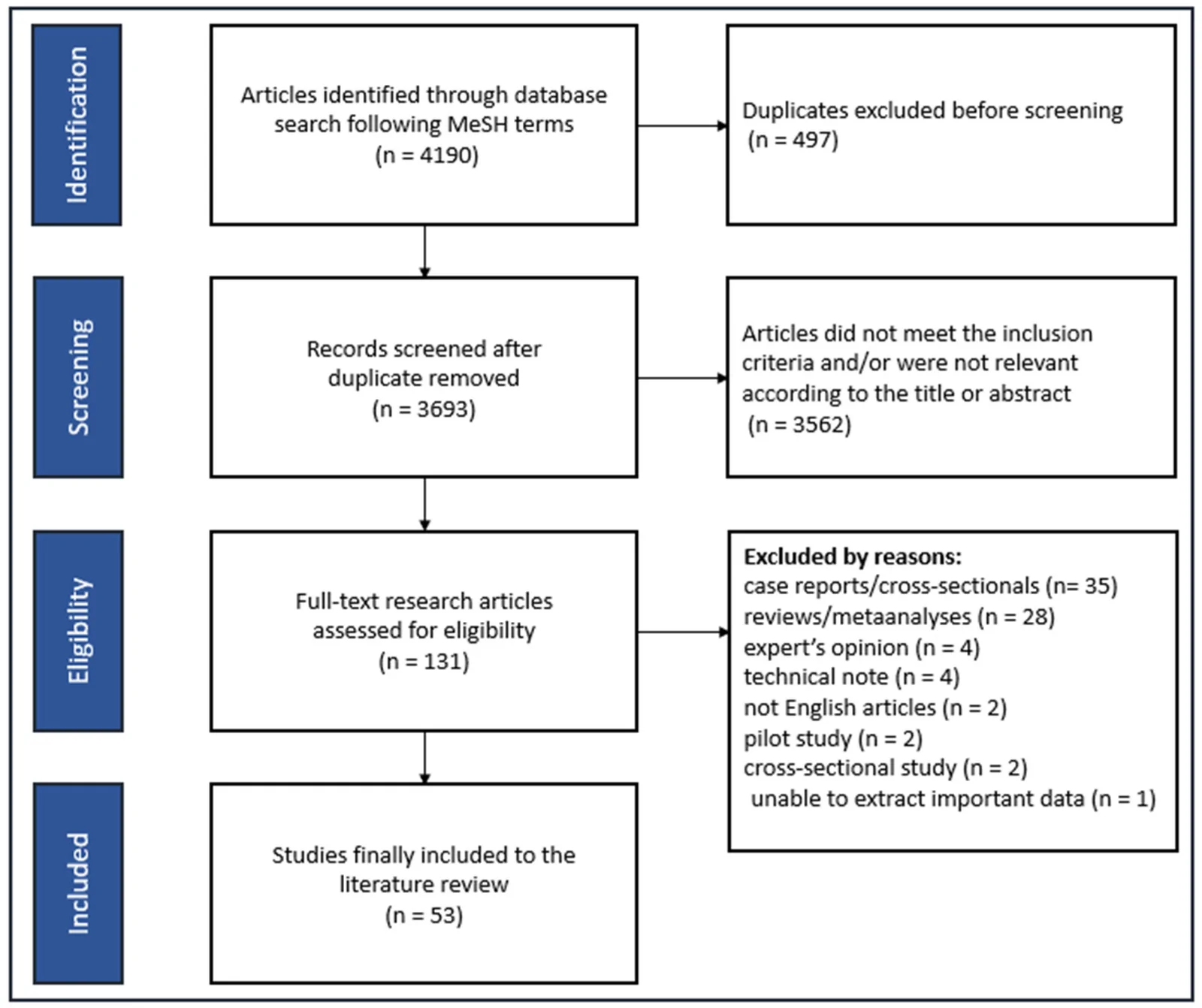
PRISMA (Preferred Reported Items for Systematic Reviews and Meta-Analyses) flow diagram of the articles extracted from the electronic databases following the key MeSH terms.

The following Medical Subject Headings (MeSH) terms “periprosthetic” and “knee” or “patella” or “tibia” and “fracture” were searched in the literature on 14 September 2025. We received 1466 results in the PubMed and 2724 results in the Science Direct database published in the period from 2015 to 2025. The review was performed independently and manually by two researchers according to the Preferred Reporting Items for Systematic Reviews and Meta-Analyses (PRISMA) guidelines (Figure 1) [13]. PRISMA checklist was added as Supplementary Table S1. The Newcastle–Ottawa scale was used check the quality of studies (Table 2) [14]. The study protocol was registered in a PROSPERO database (No. CRD420261360037). The selection criteria included periprosthetic fractures around the knee after bicondylar and not robot-assisted TKA, and their treatment methods. All available titles and abstracts were screened for the selection criteria, and full-text documents were reviewed. Case reports (reports of four cases or fewer), cross-sectional studies (given the limited value of this assessment in determining cause-and-effect relationships and the low prevalence of the disease in the population), studies offering no possibility to extract key data of patients, reviews, systematic reviews, meta-analyses, and technical notes were excluded from the analysis. Additionally, only articles in the English language and those with the full-text available were included. The following data in terms of patients’ demographics (age, gender), and clinical data (operated joint, treatment option, follow-up, and cure rate) were analyzed. The strength of evidence was assessed as moderate.

## 3. Results

### 3.1. Cohort Analysis

The median age of participants was 72 years (IQR 67.25–78). The average duration of hospitalization was 11 days (8–14), while 6 patients were hospitalized for more than 20 days. The risk of prolonged hospitalization (more than 11 days) was higher among patients with femur PPF (RR = 2.59, 95% CI 1.01–6.62). Demographic and selected clinical data are presented in Table 3.

Only 5 patients (9%) were treated conservatively. In surgical treatment, rTKA was done in 51% of cases; the remainder had fracture reduction with stabilization. The median procedure time was 100 min (IQR = 80–125). Prolonged surgery (more than 100 min) was more often associated with extended hospitalization (60.87% vs. 26.09%, *p* = 0.02). Median follow-up lasted 15 months (IQR = 7–26.5). Overall, 81% of patients were treated successfully. Coexistence of at least 1 transient disease (diabetes, obesity, hypertension, heart failure) did not affect the achievement of a cure (70.83% vs. 72.72%, *p* = 0.88). In the female group, the average time to fracture was 65.3 months, while in the male group it was 64 months. Based on patient histories, only two individuals received treatment for osteoporosis.

**Figure 2:**
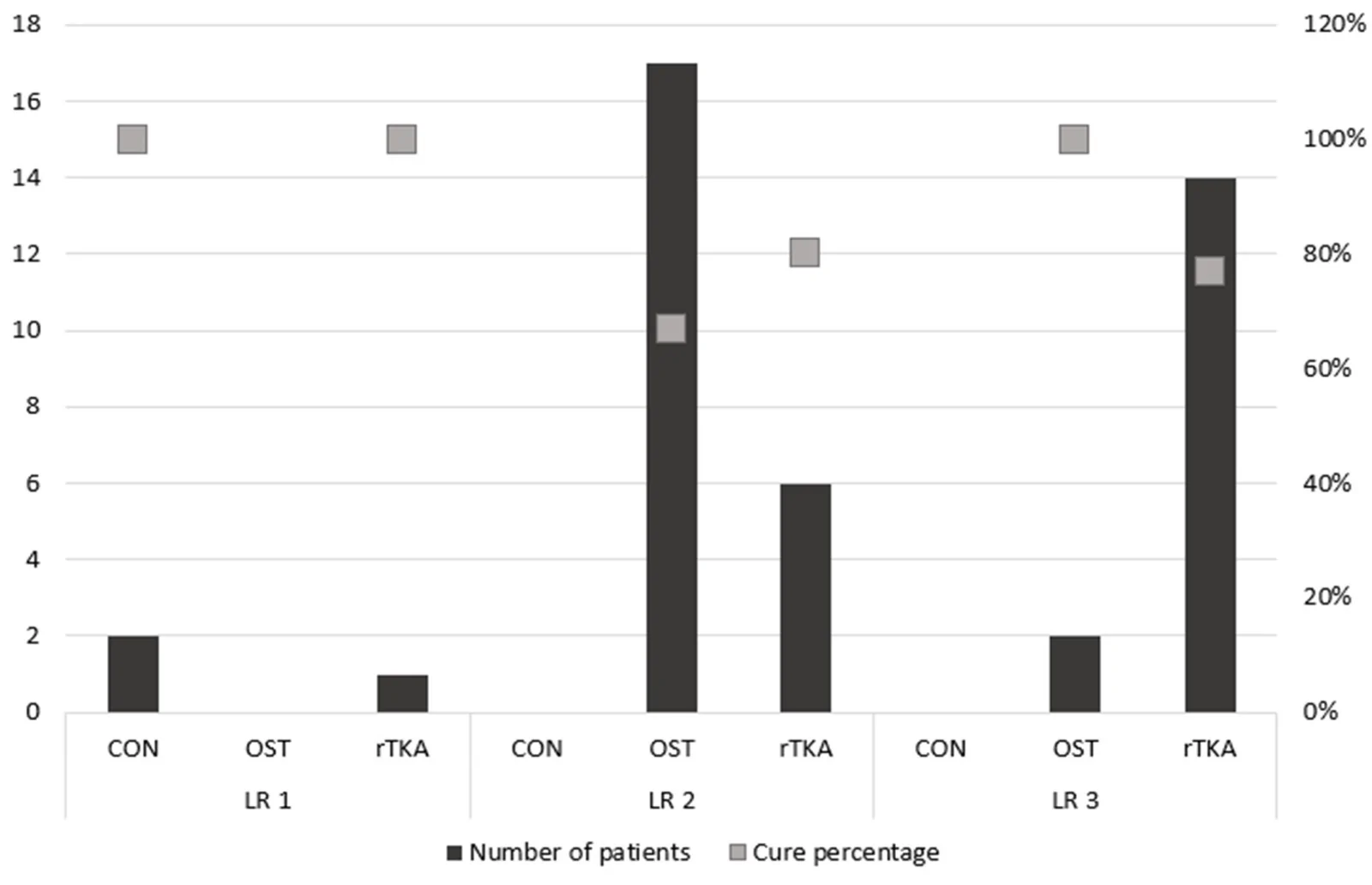
Periprosthetic fractures of the femur according to the Lewis–Rorabeck classification: treatment methods and results.

Among 42 patients with femur PPF, only 2 patients were treated conservatively, achieving fracture union (Figure 2). Replacement of the femoral component with the semi-constrained type of prosthesis was performed most frequently during rTKA (Table 4). Cable loops in the LR2 type were additionally used in 5 cases. In 16 cases, fractures were fixed with a plate during ORIF, while in 3, IMN was used. The median time for rTKA was 105 min (IQR = 85–135), while for osteosynthesis 110 min (IQR = 96.25–130.25). Overall, 69% of patients with PPF were successfully treated. The lowest treatment outcomes were observed in patients with type 2 PPF according to the LR (69%). Destabilization of the implant occurred in 4 cases after rTKA and in 5 cases with osteosynthesis (2 after LCP, and 3 after IMN). These patients required revision surgeries, but one patient died 7 days after rTKA.

Only women suffered from tibial PPF, and among them, one occurred during component implantation (Table 5). Three patients (FX1–1, FX2–1, FX3–1) were treated conservatively, and bone union was achieved in FX1 and FX2. In FX3, ORIF was performed in the revision surgery, resulting in a cure. In another case (FX2), a plate was used, but it resulted in nonunion. The remainder of the patients (FX2–1, FX3–4) received rTKA with a stemmed tibial component. Semi-constrained type implants were the preferred method of choice used in rTKA. The median operation time was 74 min (IQR = 66–78).

Just 4 patients (only women) suffered from patella fractures after TKA. In our study, there were no fractures around the patellar component, but a native patella fracture associated with a knee prosthesis. The cause of injury was a fall. Three were treated surgically with ORIF with cerclage, achieving union in all cases. The patient who was treated conservatively did not achieve fracture union and required surgery.

### 3.2. Literature Review

In the review, we included 2508 patients with femoral PPF, 186 with tibial PPF, and 29 with patellar PPF. Regardless of the applied treatment method, the overall success rate of treatment was 90% for femoral PPF, 79% for tibial PPF, and 89% for patellar PPF.

#### 3.2.1. Periprosthetic Fractures of the Femur

Only 1 patient with a post-surgical fracture (nondisplaced type 1 according to LR) and 7 patients with minor avulsion intraoperative fractures were treated conservatively (Table 6) [66]. Various surgical techniques were used in the remaining cases. Nozaka et al. [52] used a circular external fixator (EX-FIX) in the treatment of a small group of patients with comorbidities and high surgical risk, achieving a cure in all cases, and similar walking ability as before the injury in 68% of cases.

The use of LCP was the most frequent method in the treatment of femoral PPF, and an analysis of 1134 cases shows an overall cure rate of 86% [15,17,18,19,21,23,24,25,29,30,33,34,37,40,41,42,44,45,47,48,49,59,60,64,66]. This procedure is characterized by a low risk of instability and is used particularly in type 2 fractures according to LR [41]. In the study published by Caterini et al. [25], all patients returned to their pre-injury activity.

Dual plating is an alternative to standard LCP, which provides additional stabilization and is preferably used in very distal femur fractures. Of the 125 patients, 91% were successfully treated using dual plating (DP) [21,26,37,39,55,56]. Dual plating involves a larger surgical incision, but there is no significant difference in increased risk of surgical site infection (SSI) [37,64].

Intramedullary nailing is an alternative to LCP in the treatment of PPF of the femur. This is the most commonly chosen method for LR type I fractures and for some LR2 fractures. The method of choice is retrograde IMN, although the antegrade technique was also rarely used. In the group of 254 patients treated with IMN, 226 (89%) achieved bone union [15,16,21,29,31,34,40,42,48,54,60,63]. Compared to LCP, IMN is associated with higher complication risk for angular deformity and shortening of the operated limb; however, these differences are not usually significant [15,29,34,54]. The functional results (LCP versus IMN) are similar, although bone union was achieved later in the IMN group [28,40,54]. The timing of weight-bearing remains controversial. According to Maltivich et al. [14], there is no difference in the choice of treatment method, but Abboud et al. [48] points to earlier weight bearing after IMN. Use of IMN is associated with less blood loss than with LCP [29]. Arthroscopy can also be effectively used to obtain minimal access to the nail’s entry point in the femur [15]. Rarely used combinations of LCP and IMN give very good stability and result [21].

Another alternative for the treatment of femoral PPF is rTKA. It is particularly used in patients with loosened implants (LR type 3) and in complex LR type 2 cases. In the case of rTKA, the cure rate is 84% [17,18,19,27,35,41,42,44,47,59,63,66]. Complications after rTKA mainly concern SSI [47].

Leino et al. [60] emphasize that there is no difference in treatment outcomes between methods (osteosynthesis and rTKA), although nonunion occurs less frequently after ORIF than SSI after rTKA. On the other hand, rTKA is characterized by very good functional outcomes in early postsurgical time [18,33,35,44]. In turn, Battut et al. [18] indicate comparable mortality risk after rTKA and ORIF.

Excluding patients with loosened implants, rTKA is a good option for cases with poor bone quality, very distal fracture configuration, severe osteoporosis, and old age rTKA [66]. Megaprosthesis is an alternative to standard rTKA. It involves radical surgery but also gives good overall results [58]. However, according to de Marco et al. [27], ORIF appears to result in fewer complications and reoperations in comparison to megaprostheses.

#### 3.2.2. Periprosthetic Fractures of the Tibia

Six patients were treated conservatively due to very poor health conditions: five achieved union, but one required ORIF (Table 7) [20,43,53]. The most commonly used treatment method was LCP. It achieves cure rates of 83% [17,20,32,38,43,50,51,53,61,62]. In addition, MIPO improves the result to 90% successful treatment [32,38,66]. With the use of the MIPO technique, patients have a greater chance of returning to their pre-injury level of activity than traditional ORIF [20,38,62]. During ORIF, DP in tibial PPF does not offer any additional benefits and has a similar complication rate [50]. In FX type 3 fractures, IMN can be successfully inserted forward from the tibial component of the knee prosthesis, ensuring very good mechanical stability and a satisfactory cure rate [20]. External stabilization, on the other hand, does not offer promising results, with 50% of patients requiring additional surgery [43]. Revision TKA in patients with tibial PPF has very good results, and bone defects are well supplemented by the use of cones [20,53]. However, this is not the preferred method due to the required extensive surgical incision. Arthrodesis and amputation are two marginal treatment methods that have been used individually [61]. In tibial PPF, FX type 3 fractures are the most common and generally heal well, although it requires a long time to achieve bone union [43,53]. Type 1 FX fractures most often occur intraoperatively and can be treated with either rTKA or ORIF. Type 2 fractures are suitable for rTKA with long-stemmed components [20].

#### 3.2.3. Periprosthetic Fractures of the Patella

Fracture associated with the patellar implant occurs most rarely as a PPF (Table 8). Conservative treatment is a good choice in cases of non-displaced fractures or lateral edge fractures while maintaining the stability of the implant [36]. In the case of displaced fractures, the use of cerclage or a plate gives very good union results and better functionality compared to conservative treatment [15,36]. However, both injury and surgical treatment carry a 40% risk of reoperation and implant removal due to instability. In the case of treating patellar fractures following TKA without a patellar implant, the treatment follows the same protocol as for a native patellar fracture and yields good long-term outcomes [22].

## 4. Discussion

On average, PPF after TKA occurs due to low-energy trauma, such as a fall from one’s own height, as was also demonstrated in our study [19,27,45]. In the early period after the primary procedure, PPF is mostly affected by trauma or certain errors in surgical technique [66]. Taking a look at very early complications in our case, an iatrogenic fracture occurred in only one case during the insertion of the tibial component of the prosthesis and involved FX type I, which did not significantly change further treatment. Proximal tibial PPF may be caused by improper cementation or implant positioning [68]. On the other hand, analyzing femur PPF, Stamiris et al. [69] concluded that anterior notching of the femur more than 3 mm is associated with an increased risk of supracondylar femur PPF. This is due to the increase in stress on the femur during flexion, which increases as the knee bends [70]. The risk of fracture is comparable between CR and PS knee prostheses, as demonstrated biomechanically by Jethanandani et al. [71].

Also, the type of implant used can affect the risk of PPF, and it is lower in a non-CR design [70]. In rTKA, we have observed the use of implants with a higher level of constraint, such as a constrained condylar knee implant, to obtain better stability. In revisions, cones or sleeves can also be added to improve fixation in the II zone according to Morgan-Jones et al. [72]. Revision with the use of megaprosthesis for PPF may be the only viable option in some cases, excluding those associated with tumor resection [58]. Bone defects following failed TKA are the most common non-neoplastic indication for the use of a megaprosthesis [73]. The surgery involves large-scale intervention and is associated with a higher rate of complications than standard replacement surgery. The most common of these is SSI, which occurs in approximately 18% of cases. According to Vaishya et al. [73], the survival rate of the megaprosthesis alone is approximately 75% after one year, and 40% after 10 years.

The number of complications depends on the applied method of treatment and still remains a matter of discussion. The most common of these are sequentially SSI, loss of fixation stability, and malunion [74]. In comparison, the most common complication in our study was loss of fixation stability. Some studies pointed to no difference in the incidence of infection in relation to surgical strategy [14,21,60]. However, Quinzi et al. [75] indicate more frequent SSI after rTKA than LCP and IMN.

Analyzing fixations, both IMN and LCP have similar overall reoperation rates, or this difference is not statistically significant [34,41,52]. In our study, 9% of patients were reoperated on due to these complications. Further elaboration is needed on the topic, focusing on patients with femoral PPF LR type 2, as the poorest treatment results were observed in this group of patients. This suggests the need for additional plate fixation to stabilize the fracture. When analyzing PPF-type tibial fractures, we observed a healing rate similar to that reported in the systematic review. However, for Type 3 fractures according to the FX classification, rTKA with a long stem was performed, whereas the IMN method is well-established in the scientific literature, yielding very good results and representing a less invasive treatment option. As for patellar fractures without prior resurfacing surgery, they are characterized by very good treatment outcomes, and the indications for treatment correspond to those for patellar fractures in the native knee joint.

Various efforts can be made to reduce postoperative complications. In addition to adequate general preparation of the patient, the morphology of the fracture should be carefully analyzed. The current vast availability of computed tomography is essential in preoperative planning, especially in terms of assessing the stability of the endoprosthesis. Given the years of our observation, it was not used often enough in our study (in 24 cases) and may have contributed to treatment failures. In addition, operating techniques can also be modified. The use of locking plates nowadays is considered standard practice, especially in the older population, but has reasonable and proven superior results in maintaining the stability of fixation [73]. In addition, MIPO reduces the risk of nonunion and refracture compared to open methods [54,56,64,66]. Double plates should be considered in patients with fractures close to the joint, and who have risk factors for instability [21,26,37]. Another method may be the use of hybrid IMN and LCP designs, which are becoming an increasingly popular technique to avoid diseases associated with prolonged postoperative immobilization [4].

Our study has several limitations that warrant consideration. The most prominent is the relatively small sample size. Although PPF following TKA is uncommon, our study included a similar or greater number of patients than many recent publications and incorporated a systematic review to enhance its scope. It was also a retrospective study evaluating clinical outcomes conducted at two different centers over a long period of time with mid-term follow-ups. Patients were treated by several surgeons, each of whom had their own treatment philosophy. There were no hospital guidelines and protocols for treating PPF. For this reason, further detailed research on PPF after TKA is necessary, especially concerning tibial and patellar PPF. The quality of follow-up was also varied, which limits functional assessment. Another limitation stems from the heterogeneity of the study group, including variations in fracture location, bone quality, implant types, fracture patterns, and the stability of prosthesis fixation. These diverse patient and surgical factors may significantly influence treatment outcomes.

The presented study and systematic review have provided a more detailed understanding of the issue of fractures following knee arthroplasty. They have highlighted the critical need to achieve the stability of the joint during interventions. Therefore, surgeons should be encouraged to use comprehensive treatment methods that can be successfully applied in a minimally invasive manner to achieve stability, particularly in complex, near-articular epiphyseal PPF.

## 5. Conclusions

Periprosthetic fractures after TKA are rare. The complexity of the fracture and the patient’s overall condition present a significant clinical challenge in treatment planning, including computed tomography to accurately assess the stability of the prosthesis and fracture pattern. This is especially vital for PPF of the distal femur with preserved prosthesis stability. Osteosynthesis gives a good functional result. The latest minimally invasive techniques described in the literature improve treatment outcomes, which should encourage the use of these methods (e.g., MIPO) in everyday clinical practice. In rTKA, implants with a higher level of constraint are generally used to provide better stability to the knee joint.

## Figures and Tables

**Table 1:** Classification of periprosthetic fractures around the knee.

Lewis–Rorabeck classification of femur PPFs	
Type I	Nondisplaced fracture with intact prothesis component
Type II	Displaced fracture with intact prothesis component
Type III	Displaced fracture with loose prothesis component
Felix classification of tibial PPFs	
Type I	Fracture of plateau
Type II	Fracture adjacent to tibial stem
Type III	Fracture of shaft distally to component
Type IV	Fracture of tubercle

**Table 2:** Assessment of the quality of included studies according to the Newcastle–Ottawa scale.

Study	Selection				Comparability		Outcome			T1
	S1	S2	S3	S4	C1	C2	O1	O2	O3	
Abboud et al. [15]	+	+	+	+	+	+	+	-	+	8
Ali Lari et al. [16]	?	-	+	+	+	?	+	+	+	6
Atalay et al. [17]	?	?	+	+	+	?	+	+	+	6
Baron et al. [18]	+	+	+	+	+	+	+	+	+	9
Battut et al. [19]	+	+	+	+	+	?	+	+	+	8
Bauer et al. [20]	?	-	+	+	+	-	+	+	+	6
Bilodeau et al. [21]	+	+	+	+	+	+	+	?	+	8
Blum et al. [22]	+	-	+	+	+	?	+	?	+	6
Campbell et al. [23]	+	+	+	+	+	+	+	+	+	9
Canton et al. [24]	-	-	+	+	+	+	+	+	+	7
Caterini et al. [25]	?	-	+	+	+	+	+	+	+	7
Cicek et al. [26]	+	-	+	+	+	-	+	+	+	7
De Marco et al. [27]	-	+	+	+	+	+	+	+	+	8
Deshmukh et al. [28]	-	-	+	+	+	-	+	+	+	6
Ernik et al. [29]	+	+	+	+	+	+	+	+	?	8
Fink et al. [30]	?	-	+	+	+	+	+	+	+	7
Finzi et al. [31]	+	+	+	+	+	+	+	+	+	9
Fitch et al. [32]	-	-	+	+	+	+	+	+	+	7
Gan et al. [33]	+	-	+	+	+	-	+	+	+	7
Gausden et al. [34]	+	-	+	+	+	+	+	-	+	7
Girgis et al. [35]	+	-	+	+	+	+	+	+	+	8
Govil et al. [36]	-	-	+	+	+	+	+	?	+	8
Kim et al. [37]	+	+	+	+	+	+	+	+	+	9
Kim et al. [38]	+	+	+	+	+	+	+	+	+	9
Kriechlink et al. [39]	+	-	+	+	+	+	+	+	+	8
Kyriakidis et al. [40]	+	+	+	+	+	+	+	-	+	8
Labott et al. [41]	+	-	+	+	+	+	+	+	+	8
Leino et al. [42]	+	+	+	+	+	+	+	+	+	9
Liu et al. [43]	+	-	+	+	+	+	+	-	-	6
Lizcano et al. [44]	+	+	+	+	+	+	+	+	+	9
Lotzien et al. [45]	+	-	+	+	+	+	+	+	+	8
Maloti et al. [46]	+	+	+	+	+	+	+	+	+	9
Matar et al. [47]	+	-	+	+	+	+	+	+	+	8
Matlovich et al. [48]	+	+	+	+	+	+	+	?	+	8
Mazur et al. [49]	+	+	+	+	+	+	+	?	+	8
Morwood et al. [50]	+	+	+	+	+	+	+	?	+	8
Nagwadia et al. [51]	+	-	+	+	+	?	+	-	-	5
Nozaka et al. [52]	+	-	+	+	+	?	+	+	+	7
Pannu et al. [53]	+	+	+	+	+	+	+	+	+	9
Park et al. [54]	+	+	+	+	+	+	+	+	+	9
Park et al. [55]	+	-	+	+	+	+	+	?	+	7
Park et al. [56]	?	-	+	+	+	+	+	+	+	7
Pujol et al. [57]	-	-	+	+	+	-	+	+	+	6
Risitano et al. [58]	?	-	+	+	+	?	+	+	+	6
Ruder et al. [59]	+	?	+	+	+	?	+	?	+	6
Rudolph et al. [60]	+	+	+	+	+	+	+	+	?	8
Schreiner et al. [61]	+	+	+	+	+	+	+	+	?	8
Sim et al. [62]	+	+	+	+	+	+	+	+	+	9
Tandon et al. [63]	+	+	+	+	+	+	+	+	+	9
Thukral et al. [64]	+	+	+	+	+	?	+	+	+	8
Van Rysselberghe et al. [65]	+	+	+	+	+	+	+	?	+	8
Verma et al. [66]	+	-	+	+	+	+	+	-	-	7

Risk of bias: green—low, yellow—unclear, red—high. S1—Representativeness of exposed cohort S2—Selection of non-exposed cohort S3—Ascertainment of exposure S4—Demonstration that outcome of interest was not present at start of study C1—Adjust for most important risk factors C2—Adjust for other risk factors O1—Assessment of outcome O2—Follow-up length O3—Loss to follow-up rate T1—Total quality score (9 low risk of bias, 7–8 moderate risk of bias, 6 or below high risk of bias).

**Table 3:** Characteristics of the study population.

Parameter:	Female N = 49	% (88)	Male N = 7	% (12)	Total N = 56	% (100)
Age						
Below 60	4	7	1	2	5	9
61–70	13	23	3	5	16	28
71–80	24	43	1	2	25	45
81 and above	8	14	2	4	10	18
Injury						
Low-energy trauma ^1^	37	66	3	5	40	71
High-energy Trama ^2^	12	21	4	7	16	29
Comorbidities						
Arterial hypertension	26	46	5	9	31	55
Diabetes mellitus	16	35	3	5	19	34
Gout/hiperurykemia	1	2	3	5	4	7
Heart failure	3	5	1	2	3	5
Obesity	10	18	2	4	12	21
Rheumatoid arthritis	2	4	0	0	2	3
Fracture localization						
*Femur*	*35*	*63*	*7*	*13*	*42*	*75*
LR type I	2	4	1	2	3	4
LR type II	19	34	4	7	23	41
LR type III	14	25	2	4	16	30
*Tibia*	10	18	0	0	10	18
FX type I	1	2	0	0	1	2
FX type II	4	7	0	0	4	7
FX type III	5	9	0	0	5	9
*Patella*	4	7	0	0	4	7

^1^-fall from own height or bed, ^2^-fall from stairs or bicycle.

**Table 4:** Types of knee endoprostheses used primarily and during revision arthroplasty.

Primary Used Implant Type	Number	%	Implant Type Used During rTKA	Number	%
Cruciate retaining	7	33	Cruciate retaining	3	14
Posterior stabilized	12	57	Posterior stabilized	6	29
Condylar constrained	2	10	Condylar constrained	8	38
			Rotating hinge or global modular	4	19

rTKA—revision total knee arthroplasty.

**Table 5:** Periprosthetic fractures of the tibia according to the Felix classification: treatment methods and results.

Fracture Type	Patients		Treatment Method				Cure Rate	
	N	%	Conservative	%	Surgery	%	N	%
FX I	1	10	1	100	0	0	1	100
FX II	4	40	1	25	3	75	3	75
FX III	5	50	1	20	4	80	5	100

FX—Felix classification.

**Table 6:** General overview of the analyzed studies regarding femoral PPF after TKA.

Authors	Number of Patients	Mean Age	Classification System	Treatment Method (Patients)	Cure Rate (%)
Abboud et al. [15]	438	75	SOFCOT	LCP (365) IMN (69)	LCP 89% IMN 90%
Ali Lari et al. [16]	16	71	Rorabeck	IMN	100%
Atalay et al. [17]	15	69	Rorabeck	LCP	100%
Baron et al. [18]	62	81.2	SOFCOT	LCP (18) rTKA (44)	89%
Battut et al. [19]	52	80.5	SOFCOT	LCP (20) rTKA (32)	LCP 52% rTKA 74%
Bilodeau et al. [21]	78	79	AO/OTA	LCP (21) DP (17) LCP triple plate (4) LCP + IMN (24) IMN (13)	LCP one plate 78% LCP dual or triple plate 76% LCP + IMN 91% IMN 85%
Campbell et al. [23]	55	76	Rorabeck	LCP	89%
Canton et al. [24]	19	84	Rorabeck	LCP	92%
Caterini et al. [25]	12	78	Rorabeck	LCP	100%
Cicek et al. [26]	22	73	Su	DP	91%
De Marco et al. [27]	13	73	Rorabeck	LCP (9) rTKA (4)	89% LCP 50% rTKA
Deshmukh et al. [28]	16	71	Su	rTKA	88%
Ernik et al. [29]	32	79	Rorabeck	LCP (20) IMN (12)	90% LCP 100% IMN
Fink et al. [30]	15	72	-	rTKA	73%
Finzi et al. [31]	13	84	Rorabeck	IMN	100%
Gan et al. [33]	15	77	Rorabeck	rTKA (7) LCP (8)	100% rTKA 75% LCP
Gausden et al. [34]	97	76	Su	LCP (73) IMN (24)	84%
Girgis et al. [35]	14	82	Su	rTKA	79%
Kim et al. [37]	30	73	Su	LCP or DP	98%
Kriechlink et al. [39]	15	78	Su	LCP or DP	93%
Kyriakidis et al. [40]	60	79	Rorabeck	LCP (31) IMN (29)	87% LCP 93% IMN
Labott et al. [41]	56	77.2	AO	LCP (43) rTKA (13)	95% LCP 65% rTKA
Leino et al. [42]	68	79	Rorabeck	LCP (29) rTKA (29)	75% ORIF 70% rTKA
Lizcano et al. [44]	99	74	Su	LCP (45) rTKA (54)	76% LCP 87% rTKA
Lotzien et al. [45]	45	74	Rorabeck	LCP	96%
Maloti et al. [46]	56	77	Su	LCP	88%
Matar et al. [47]	30	81	Rorabeck	rTKA	93%
Matlovich et al. [48]	57	74	Rorabeck	LCP (38) IMN (19)	100% LCP 89% IMN
Mazur et al. [49]	36	71	Su	LCP	83%
Nagwadia et al. [51]	29	64	Rorabeck	LCP	93%
Nozaka et al. [52]	11	79	Rorabeck	EX-FIX	100%
Park et al. [54]	41	72	AO/OTA	IMN (20) MIPO (21)	85% IMN 95% MIPO
Park et al. [55]	21	76	Su	DP	95%
Park et al. [56]	18	75	Rorabeck	MIPO + DP	100%
Pujol et al. [57]	11	77	Rorabeck	rTKA	64%
Rahman et al. [67]	17	76	Rorabeck	rTKA	88%
Risitano et al. [58]	9	82	Rorabeck	Mega-rTKA	89%
Ruder et al. [59]	58	80	-	LCP (35) rTKA (23)	LCP 91% rTKA 94%
Rudolph et al. [60]	59	78	Su	LCP (41) IMN (22)	LCP 61% IMN 63%
Tandon et al. [63]	61	76	Kim	LCP (23) IMN (17) rTKA (21)	80% LCP + IMN 100% rTKA
Thukral et al. [64]	31	72	Rorabeck	LCP (17) MIPO (14)	94% LCP 100% MIPO
Van Rysselberghe et al. [65]	295	75	OTA	LCP (224) IMN (71)	LCP 84% IMN 92%
Verma et al. [66]	38	66	Rorabeck	CON (8) LCP (26) rTKA (4)	100% CON 70% LCP 100% rTKA

SOFCOT-French Society of Orthopaedic and Trauma Surgery Classification, AO-Association of the Study of Internal Fixation classification, OTA-Orthopaedic Trauma Association classification, CON—conservative treatment, IMN—intramedullary nailing, LCP—low compression plate, Mega-rTKA—megaprosthesis, MIPO—minimally invasive plate osteosynthesis, rTKA—revision knee arthroplasty.

**Table 7:** General overview of the analyzed studies regarding tibial PPF after TKA.

Authors	Number of Patients	Mean Age	Classification System	Treatment Method (Patients)	Success Rate of Treatment (%)
Atalay et al. [17]	5	69	Felix	ORIF	60%
Bauer et al. [20]	15	72	SOFCOT	CON (7) LCP (60) IMN (13) rTKA (20)	100% CON 18% LCP 100% IMN 100% rTKA
Fitch et al. [32]	8	68	Felix	LCP	88%
Kim et al. [38]	16		Felix	MIPO	88%
Liu et al. [43]	24	72	Felix	CON (1) EX-FIX (14) LCP (8) Spacer (1)	0% CON 50% EX-FIX 63% LCP 100% Spacer
Morwood et al. [50]	38	68	Felix	LCP	76%
Nagwadia et al. [51]	29	64	Felix	LCP	100%
Pannu et al. [53]	34	68	Felix	CON (5) LCP (16) rTKA (7)	CON 100% LCP 63% rTKA 29%
Schreiner et al. [61]	9	77	Felix	LCP (6) rTKA (1) Artrodesis (1) Amputation (1)	LCP 33% rTKA 100% Artrodesis 100%
Sim et al. [62]	18	74	Felix	LCP	100%
Verma et al. [66]	4	66	Felix	MIPO	100%

SOFCOT-French Society of Orthopaedic and Trauma Surgery Classification, CON—conservative treatment, EX-FIX—external fixator, IMN—intramedullary nailing, LCP—low compression plate, MIPO—minimally invasive plate osteosynthesis, rTKA—revision knee arthroplasty.

**Table 8:** General overview of the analyzed studies regarding patellarPPF after TKA.

Authors	Number of Patients	Mean Age	Classification System	Treatment Method (Patients)	Success Rate of Treatment (%)
Blum et al. [22]	15	65	-	OST	87%
Govil et al. [36]	6	64	Goldberg	CON (2) OST (4)	100% CON 100% OST
Nagwadia et al. [10]	5	66	Goldberg	OST	80%
Verma et al. [66]	4	66	Goldberg	CON (1) OST (4)	100% CON 100% OST

CON—conservative treatment, OST—osteosynthesis.

## Data Availability

The datasets used and/or analyzed during the current study are available from the corresponding author on reasonable request.

## Supplementary Materials

The following supporting information can be downloaded at: https://www.mdpi.com/article/10.3390/medicina62091727/s1, Supplementary Table S1: PRISMA 2020 checklist.

## Abbreviations

The following abbreviations are used in this manuscript:

AP	anteroposterior
BMI	body mass index
CI	confidence interval
CT	conservative treatment
CRIF	close reduction and internal fixation
DP	dual plating
EX-FIX	external fixator
FX	Felix classification
IMN	intramedullary nailing
IQR	interquartile range
LCP	low compression plate
LR	Lewis–Rorabeck classification
MIPO	minimally invasive plate osteosynthesis
MeSH	Medical Subject Headings
ORIF	open reduction and internal fixation
PF	periprosthetic fractures
RR	risk ratio
SSI	surgical site infection
rTKA	revision total knee arthroplasty
TKA	total knee arthroplasty
